# Preliminary evaluation of probiotic effects on gastrointestinal signs in dogs with multicentric lymphoma undergoing multi‐agent chemotherapy: A randomised, placebo‐controlled study

**DOI:** 10.1002/vro2.2

**Published:** 2021-03-29

**Authors:** Maria C. Jugan, Raelene M. Wouda, Mary Lynn Higginbotham

**Affiliations:** ^1^ Department of Clinical Sciences College of Veterinary Medicine Kansas State University Manhattan Kansas USA

**Keywords:** canine, chemotherapy toxicity, CHOP protocol, dysbiosis, probiotics

## Abstract

**Background:**

Gastrointestinal (GI) toxicity is a major dose‐limiting factor in dogs undergoing chemotherapy. A proposed mechanism of GI toxicity includes chemotherapy‐driven GI dysbiosis. This study was designed to determine the effects of probiotic administration on GI side‐effects in dogs receiving multi‐agent chemotherapy.

**Methods:**

Ten client‐owned dogs with multicentric lymphoma were enrolled in a prospective, randomised, placebo‐controlled single‐blinded study. On the first day of the cyclophosphamide doxorubicin vincristine prednisone (CHOP)‐based chemotherapy protocol, dogs were randomised to receive either daily oral probiotic at a dose of 200 × 10^9^ cfu/10 kg (n = 5) or daily oral placebo (n = 5). Complete blood count, faecal score (FS), faecal microbiome analysis (qPCR) and adverse events scores were performed at baseline and on the day of each subsequent chemotherapy dose, as well as 3 days after doxorubicin (days 0, 7, 14, 21, 24 and 28).

**Results:**

Overall, 40% of dogs had an abnormal GI microbiome at baseline, specifically decreased faecal *C. hiranonis* and *Fusobacterium* abundances. Dogs receiving probiotics had increased faecal *Streptococcus* (p = 0.02) and *E. coli*. (p = 0.01). No dogs receiving probiotics experienced diarrhoea (FS ≥ 3.5) compared to four of five receiving placebo. (F 2.895; p = 0.13)

**Conclusion:**

GI microbiome dysbiosis was common in this group of dogs with multicentric lymphoma. Probiotics were well‐tolerated, with no negative side effects. Further studies are needed to explore broader microbiome and metabolome changes, as well as clinical benefit.

**Abbreviations**
cfu: colony‐forming units
CHOP: cyclophosphamide doxorubicin vincristine prednisone
DI: dysbiosis index
FS: faecal score
GI: gastrointestinal
IBD: inflammatory bowel disease
PARR: PCR for antigen receptor rearrangement
VCOG‐CTCAE: veterinary cooperative oncology group common terminology for adverse events

## INTRODUCTION

Chemotherapy‐induced gastrointestinal (GI) toxicity is a serious complication of cancer treatment in human and veterinary patients. GI toxicity affects 15–40% of people undergoing standard‐dose chemotherapy and almost 100% undergoing high‐dose chemotherapy.^1^ In a large study of dogs undergoing multi‐agent chemotherapy for lymphoma, 40% experienced anorexia, 27% experienced vomiting and 22% experienced diarrhoea.^2^ Although doxorubicin is commonly associated with GI toxicity, recent studies demonstrated high toxicity rates with other chemotherapeutics.^3^ Dose‐limiting GI toxicities often occur 3–5 days after chemotherapy administration, and implications include increased infection risk, need for hospitalisation and need for chemotherapy dose reduction associated with decreased survival.^1^, ^4^

Changes in GI microbial populations and secondary structural GI changes, altered bacterial metabolic by‐products and increased GI inflammatory cytokines are proposed mechanisms of chemotherapy‐induced GI toxicity.^4^, ^5^ Human and rodent models evaluating probiotics as treatment or prevention of chemotherapy‐induced GI toxicity have demonstrated positive results. In paediatric patients, concurrent probiotic administration decreased febrile episodes, maintained normal faecal organic acids and decreased the need for antibiotics.^6^ Following chemotherapy‐induced GI villus shortening, rodent models have demonstrated increased villus length, reduced diarrhoea severity and decreased GI inflammatory cytokines with probiotic administration.^7^, ^8^ Importantly, rodent studies have not documented probiotic species GI translocation,^8^ a concern of administration to immunosuppressed patients.

To the authors’ knowledge, use of probiotics for prevention or treatment of chemotherapy‐induced GI toxicity in dogs has not been evaluated. Therefore, this study was designed to evaluate effects of a multi‐strain probiotic on GI clinical signs in dogs with lymphoma undergoing cyclophosphamide doxorubicin vincristine prednisone (CHOP)‐based chemotherapy. The hypothesis was that probiotic administration would decrease clinical signs of GI toxicity compared to placebo. A secondary objective was to preliminarily explore probiotic effects on the GI microbiome in this population.

## MATERIALS AND METHODS

### Study population

Ten client‐owned dogs presenting to Kansas State University Veterinary Health Center were enrolled prospectively with informed consent following a diagnosis of multicentric, non‐GI lymphoma and prior to their first chemotherapy dose. Lymphoma was diagnosed via cytology or histopathology by a board‐certified veterinary pathologist. Flow cytometry and PCR for antigen receptor rearrangement (PARR) were performed to confirm the diagnosis of lymphoma, as needed. Staging minimally included a CBC and serum biochemistry panel, with additional testing at the discretion of the attending clinician. Exclusions were historical GI signs, a novel antigen/hydrolysed diet to control historical GI signs, ultrasonographic abnormalities of the GI tract and antibiotics within 1 month or anti‐emetics within 1 week of initial chemotherapy. Dogs undergoing chemotherapy protocols other than CHOP were excluded. The Institutional Animal Care and Use Committee at Kansas State University approved the study protocol (#4276).

### Study design

A randomised, placebo‐controlled, single‐blinded study was performed. Dogs were assigned to one of two treatment groups by a non‐blinded investigator (Maria C. Jugan) via simple randomization (random number generator, odd versus even) until five dogs were included in each group. Dogs received either a multi‐strain probiotic (112.5 colony‐forming units [cfu]/capsule; *Streptococcus thermophiles, Bifidobacterium breve, B. longum, B. infantis, Lactobacillus acidophilus, L. plantarum, L. paracasei, L. delbrueckii bulgaricus*) (Visbiome; ExeGi Pharma, LLC, Rockville, MD, USA) at dose of 200 × 10^9^ cfu/10 kg or placebo (maltodextrin in gelatin capsules), with the number of capsules equivalent to probiotic dose, once daily on food for the duration of the study.^9^ Number of capsules was rounded to the nearest full‐capsule dose. Capsules were refrigerated by owners, opened, and contents given on the dog's normal food. Owners and investigators performing recheck examinations (Raelene M. Wouda and Mary Lynn Higginbotham) were blinded to treatment group. Dogs received chemotherapy treatments on days 0 (vincristine), 7 (cyclophosphamide), 14 (vincristine) and 21 (doxorubicin), with chemotherapy dose at the attending clinician's discretion. Probiotic/placebo treatment was started after the first chemotherapy dose and continued through day 28 or 1 week past doxorubicin in dogs with dose delays. Anti‐emetics and antibiotics were not prophylactically administered at home following chemotherapy but were allowed if deemed necessary by the attending clinician.

Faecal samples (naturally voided or collected via digital rectal examination) and blood for CBC were obtained on days 0, 7, 14, 21 and 28 prior to the scheduled chemotherapy, as well as 3 days after doxorubicin (day 24) to allow sample collection when GI side‐effects are most commonly observed. When dose delays occurred, samples were obtained on the next day of chemotherapy. Faecal samples were either collected in‐hospital and stored at −80⁰C within 4 hours or collected at home at the time of defaecation, shipped overnight on ice, and stored at −80⁰C on receipt. CBC was performed within 24 hours of collection using an in‐house commercial analyser (Advia 120 Hematology System; Siemens HealthCare, Munich, Germany). Faecal microbiome analysis via qPCR for total bacteria and seven taxa (*Faecalibacterium spp, Turicibacter spp, Escherichia coli, Streptococcus spp, Blautia spp, Fusobacterium spp, Clostridium hiranonis*) and subsequent calculation of the dysbiosis index (DI) were performed through the Texas A&M Gastrointestinal Laboratory as previously described.^10^ The DI is reported as a unitless value representing deviation of the diseased dog's microbiome from that of healthy dogs based on the combined qPCR analyses. The DI was derived from a large number of healthy dogs across varied environments and with varied diets,^10^ allowing these variables to be accounted for in a study population.

**FIGURE 1:**
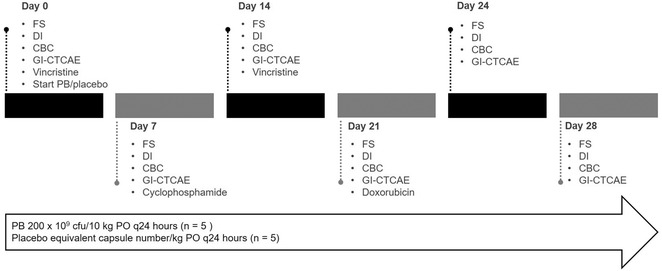
Study timeline for 10 dogs undergoing CHOP‐based chemotherapy for multicentric lymphoma and receiving either probiotic or placebo Abbreviations: CBC, complete blood count; cfu, colony forming units; DI, dysbiosis index; FS, faecal score; GI‐CTACAE, Gastrointestinal Veterinary Cooperative Oncology Group common terminology for adverse events; PB, probiotic.

Owners maintained a daily journal documenting administration of study treatment, faecal score (FS),^11^ activity, appetite, vomiting and hypersalivation. For the purposes of this study, FS ≥ 3.5 was considered diarrhoea. On days 0, 7, 14, 21, 24 and 28, owners completed a standardised questionnaire (Supplement 1), and scoring for GI toxicity was performed using the Veterinary Cooperative Oncology Group common terminology for adverse events (VCOG‐CTCAE) guidelines version 1.1.^12^ Diet was not standardised; diet was recorded, and owners were instructed not to change diets during the study. Treatment needed for chemotherapy‐related GI side effects was recorded. Figure 1 outlines the study timeline.

### Statistical analysis

Statistical analysis was performed using commercial software (GraphPad Prism Version 8.4.3; SPSS Version 27). Data were assessed for normality using the Shapiro‐Wilk test and expressed as mean ± SD for normal data or median (range) for non‐normal data. Significance was set at p < 0.05.

Baseline DI and faecal bacteria taxa were compared between the study population and laboratory reference range using descriptive statistics. Baseline dog characteristics, FS, DI values and faecal bacteria abundances (log DNA/gram faeces) were compared between dogs receiving probiotic versus placebo using the unpaired t test or Mann‐Whitney U test.

To evaluate effects of probiotic versus placebo on FS, GI‐CTCAE, DI and faecal bacteria taxa, a mixed analysis of variance accounting for within‐subject effects (time, interaction of treatment x time) and between‐subjects effects (treatment [i.e., probiotic]) was performed (F statistic). Greenhouse‐Geisser‐adjusted p values were used, as needed.

For descriptive comparison, DI > 2 was considered abnormal, 0–2 equivocal and <0 normal.^10^ For statistical comparison, an equivocal DI value was considered normal. FSs were analysed based on average daily FS since the previous chemotherapy dose, as well as number of days with FS 3.5, 4.0–4.9 and 5.

For variables with no significant treatment or time effect, the Wilcoxon signed rank test was used to compare values before and after chemotherapy in all dogs between recheck time‐points (eg, day 0 vs 7), accounting for carryover effects.

## RESULTS

### Study population

Ten dogs with multicentric lymphoma (n = 5 probiotic; n = 5 placebo) were enrolled and completed the study between June 2019 and May 2020. Breeds included Golden Retrievers (n = 2 probiotic), Labrador Retrievers (n = 2 placebo), Jack Russell Terriers (n = 1 probiotic; n = 1 placebo) and one each of Miniature Poodle (placebo), Heeler (placebo), Australian Shepherd (probiotic) and Scottish Terrier (probiotic). There were five spayed females (n = 3 probiotic; n = 2 placebo), two intact females (n = 2 probiotic) and three neutered males (n = 3 placebo). Mean age was 7.2 years (range, 4–13 years) for dogs receiving probiotics and 10.6 years (range, 6–14 years) for dogs receiving placebo. Mean weight and body condition score were 19.6 +/− 11.77 kg (range, 6.6–30.9 kg) and 6.4/9 (range, 5.5–7; ideal = 5/9) for dogs receiving probiotics and 24.8 +/− 15.79 kg (range, 5.3–37.9 kg) and 6.3/9 (range, 5–7) for dogs receiving placebo. There were no differences in age, weight or body condition between treatment groups.

### Lymphoma classification

The diagnosis of lymphoma was obtained via peripheral lymph node cytology (n = 7), peripheral lymph node histopathology (n = 2) and cytology of the liver and abdominal effusion in one dog in which the diagnosis was not obtained via lymph node cytology. Lymphoma in all dogs was deemed high‐grade based on combined clinical, cytologic and/or histologic characteristics. Based on cell size, lymphoma was classified as large cell in five dogs (n = 2 probiotic; n = 3 placebo) and medium cell in five dogs (n = 3 probiotic; n = 2 placebo). Immunophenotyping via flow cytometry was performed in six dogs. PARR was performed in two dogs. Lymphoma was classified as B cell in five dogs (n = 2 probiotic; n = 3 placebo), T cell in three dogs (n = 2 probiotic; n = 1 placebo) and undefined in two dogs that did not have flow cytometry or PARR (n = 1 each group).

While complete staging was not performed in all dogs, a subset of dogs had imaging performed, and all dogs had a CBC performed prior to chemotherapy. Two dogs had baseline thoracic radiographs, with no abnormalities noted. Two dogs, including one that had thoracic radiographs and one additional dog, had baseline abdominal ultrasound performed. Findings included hepatic hypoechogenicity, mottled splenic echotexture and enlarged intraabdominal lymph nodes (2.4–3.2 cm) in one dog and diffuse hepatomegaly with hypoechogenicity and mottled echotexture, diffusely hypoechoic intraabdominal lymph nodes (0.8–3.6 cm) and moderate anechoic peritoneal effusion in the other dog. Based on generalized peripheral lymphadenopathy, most dogs were classified as having at‐minimum stage 3a lymphoma (n = 5 probiotic; n = 4 placebo). One dog (placebo) had at‐minimum stage 4b lymphoma based on hepatic involvement. Bone marrow cytology and histopathology were not performed in any dog, so stage 5 disease could not be completely excluded. However, cytologic evaluation of peripheral blood at baseline was not suggestive of bone marrow involvement in any dog (i.e., lack of abnormal circulating cells and absence of bi‐ or pancytopenia). There was no difference in lymphoma stage between treatment groups.

Individual dog characteristics are summarized in Supplement 2.

### Ancillary treatments

#### Probiotic group

Two dogs experiencing ≥grade 3 neutropenia were administered a 1‐week course of amoxicillin clavulanate (13.4–17.2 mg/kg PO twice daily). Amoxicillin clavulanate was administered the week prior to cyclophosphamide (i.e., day 14 sample; n = 1) and the week prior to doxorubicin (i.e., day 21 sample; n = 1). No dogs required treatment for chemotherapy‐related GI side effects.

#### Placebo group

Three dogs required treatment for chemotherapy‐related GI side effects. One dog was administered a 2‐day course of maropitant citrate (Cerenia; Zoetis) (2.4 mg/kg PO once daily) and metronidazole (13.1 mg/kg PO twice daily) due to vomiting and diarrhoea 3 days post‐cyclophosphamide (days 17–18). A second dog received a 7‐day course of maropitant citrate (1.6 mg/kg PO once daily) and metronidazole (13.3 mg/kg PO twice daily) due to diarrhoea following cyclophosphamide (days 15–21). The third dog received metronidazole (11.3 mg/kg PO twice daily) throughout the study due to development of diarrhoea 1 day after the first dose of vincristine (days 2–28); the owner declined metronidazole discontinuation after diarrhoea resolved. No dogs required fluids or hospitalisation due to GI side effects.

### Faecal characteristics

**FIGURE 2:**
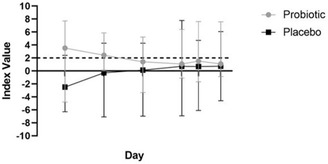
Dysbiosis index values, 10 dogs undergoing CHOP‐based chemotherapy for multicentric lymphoma and receiving either probiotic or placebo. An index value of <0 is considered normal. Dashed line indicates the dysbiosis index value, at or above which was considered abnormal Abbreviation: CHOP, cyclophosphamide doxorubicin vincristine prednisone.

**FIGURE 3:**
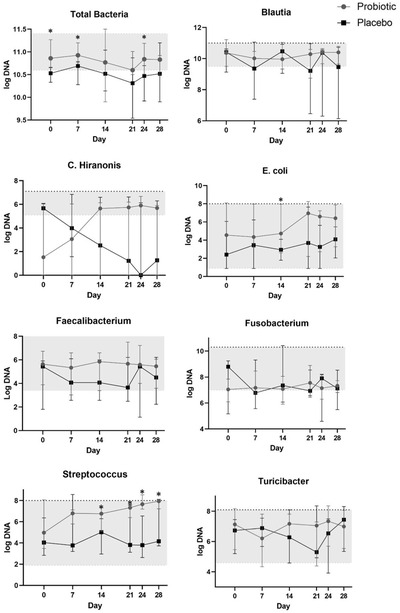
Median faecal bacteria abundances (log DNA/g feces) in 10 dogs undergoing CHOP‐based chemotherapy for multicentric lymphoma and receiving either probiotic or placebo. Shaded area denotes normal reference range Abbreviation: CHOP, cyclophosphamide doxorubicin vincristine prednisone. *p < 0.05.

Figures 2 and 3 summarise faecal DI values and bacterial abundances for the study population at baseline and between treatment groups across the course of the study, respectively.

#### FSs

**FIGURE 4:**
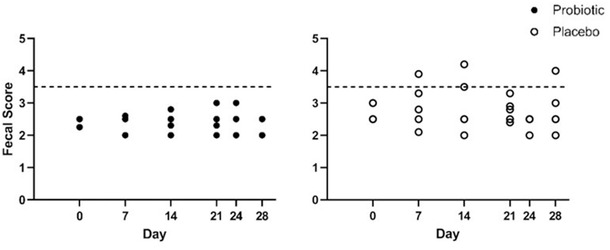
Average faecal scores since previous chemotherapy dose in 10 dogs undergoing a CHOP‐based protocol treatment for multicentric lymphoma and receiving either probiotic or placebo. The dashed line at 3.5 denotes the faecal score cut‐off, at or above which was considered diarrhoea Abbreviation: CHOP, cyclophosphamide doxorubicin vincristine prednisone.

Median baseline FS was 2.5/5 (2–3), with no difference between treatment groups (p = 0.28). There was no significant difference in FS before/after chemotherapy (p = 0.60). Although not statistically significant, no dogs receiving probiotics had a FS ≥ 3.5 at any point, while four dogs receiving the placebo had a FS ≥ 3.5 at least one time during the study, accounting for 32 total days where diarrhoea was documented across the group (F 2.895; p = 0.127) (Figure 4).

#### Faecal DI

Four of 10 dogs had evidence of GI dysbiosis at baseline, based on a DI value >2 (median −0.40 [range, −4.8–7.7]) (n = 3 probiotic; n = 1 placebo). Baseline DI was not different between groups (probiotic 3.52 [−4.8 ‐ 7.71] versus placebo −2.49 [−6.3 − 2.4]) (p = 0.42).

Of dogs with an abnormal baseline DI, two remained abnormal (n = 1 probiotic; n = 1 placebo), and one (probiotic) became equivocal during the study. Of dogs with a normal baseline DI, one (probiotic) remained normal, and three (n = 1 probiotic; n = 2 placebo) became equivocal. One dog (placebo) with an equivocal baseline DI became abnormal (Figure 2). There was no difference in DI between treatment groups at any time‐point. (F 0.876; p = 0.377)

#### Faecal microbiome

Baseline faecal total bacteria were higher in the probiotic (10.86) versus placebo group (10.53; p = 0.02) and remained higher in dogs treated with probiotic versus placebo throughout the study duration (F 8.63; p = 0.02).

There were no differences in individual faecal bacterial abundances between treatment groups at baseline. Over the course of the study, faecal *Streptococcus* (F 8.90; p = 0.02) and *E. coli* (F 12.51; p = 0.01) were higher in dogs receiving probiotic versus placebo, with no effect of time. There were no differences in faecal *Faecalibacterium*, *Turicibacter*, *Blautia*, *Fusobacterium* or *C. hiranonis* between treatment groups (Figure 3). *Streptococcus* was higher in dogs at day 21 (6.03 [3.12–7.60]) compared to day 14 (5.49 [2.96–7.35]) following the second dose of vincristine, regardless of treatment group (p = 0.047).

### Adverse effects and clinical monitoring

There were no adverse effects attributed to either the probiotic or placebo. All adverse events were either chemotherapy‐induced GI or haematologic. There was no difference in GI‐CTCAE score between treatment groups. (F 0.30; p = 0.61) (Table 1).

**TABLE 1:** Gastrointestinal side‐effects expressed as a number of days and (number of dogs) in 10 dogs undergoing CHOP‐based chemotherapy for multicentric lymphoma and receiving either probiotic or placebo. Gastrointestinal severity scores are expressed as the average score per time period. Superscript letters denote an individual dog

		Treatment	
Clinical sign	Time‐period	Probiotic	Placebo
Vomiting	Days 0–7	0	1 (1^a^)
	Days 8–14	0	1 (1^b^)
	Days 15–21	0	2 (2^c,d^)
	Days 22–28	1 (1^e^)	0
Diarrhea (FS 3.5–3.9)	Days 0–7	0	1 (1^a^)
	Days 8–14	0	5 (2^a,b^)
	Days 15–21	0	6 (2^a,c^)
	Days 22–28	0	1 (1^a^)
FS 4–4.9	Days 0–7	0	3 (2^a,b^)
	Days 8–14	0	1 (1^a^)
	Days 15–21	0	2 (2^a,d^)
	Days 22–28	0	6 (2^a,b^)
FS ≥ 5.0	Days 0–7	0	3 (1^a^)
	Days 8–14	0	4 (1^b^)
	Days 15–21	0	0
	Days 22–28	0	0
GI‐CTCAE	Days 0–7	0.33	1.2
	Days 8–14	0	1.0
	Days 15–21	0	1.0
	Days 22–28	0.2	0.4

Abbreviations: FS, faecal score; GI‐CTCAE, gastrointestinal common terminology for adverse events

Over the entire study, there were six documentations of neutropenia, including two occurrences of grade 1 neutropenia following vincristine (n = 2 probiotic), two occurrences of grade 2 neutropenia following vincristine in the same dog (placebo), one occurrence of grade 3 neutropenia following vincristine (probiotic) and one occurrence of grade 4 neutropenia following vincristine (probiotic).

## DISCUSSION

In this prospective, single‐blinded, placebo‐controlled study, GI microbiome shifts and chemotherapy‐induced GI side effects were compared in dogs with multicentric lymphoma receiving either an oral probiotic or placebo while undergoing CHOP chemotherapy. While previous studies have documented microbiome alterations in dogs with multicentric and GI lymphoma, to the authors’ knowledge, this is the first study evaluating microbiome changes related to chemotherapy or impact of concurrent probiotics. In this population, GI dysbiosis was observed at the time of diagnosis, and both chemotherapy and probiotic supplementation affected the microbiome.

Probiotics were well‐tolerated in this population, and no obvious side effects of administration were observed. While not statistically significant, dogs receiving probiotics tended to have fewer episodes of diarrhoea versus dogs receiving placebo. Two dogs in the placebo group experienced liquid diarrhoea for 7 days total during the study; both of these dogs and one other dog had 12 additional total days during which stool consistency lost form, although still had solid material. All three dogs required metronidazole due to diarrhoea; no dogs in the probiotic group required antibiotics due to GI side‐effects. Interestingly, neither of the two dogs in the probiotic group that received amoxicillin clavulanate experienced antibiotic‐induced diarrhea, a common side effect of that drug^13^; no dogs in the placebo group received amoxicillin clavulanate for comparison. The observed differences between groups suggest a potential benefit of the probiotic, with lack of significant difference likely due to low statistical power.

At the time of lymphoma diagnosis, four of 10 dogs had evidence of GI dysbiosis based on an increased DI value, with an additional dog having an equivocal value. This percentage is similar to previous work comparing the DI in dogs with multicentric lymphoma to healthy dogs.^14^ The DI value changed during the study in several dogs, including three dogs with a normal baseline DI that had an equivocal value at completion and one dog with an equivocal baseline value that had an abnormal value at completion. However, the DI tended to decrease (improve) in dogs receiving probiotics and increase in dogs receiving placebo; this was not statistically significant. The combination of an increased DI trend in the overall population paired with an improving DI in the probiotic group could suggest negative impact of chemotherapy and possible protective probiotic effect. However, consideration of the dog's disease status and adjunctive treatments (eg, antibiotics), as well as larger population size to determine clinical relevance would be needed.

Three of 10 dogs in this study also had increased faecal total bacteria at diagnosis. Increased total bacteria may be due to small increases in several taxa enumerated or populations not assessed by targeted analysis. At diagnosis, dysbiosis was characterised by decreased *C. hiranonis* and *Fusobacterium* compared to expected abundances in healthy dogs. While decreased *Fusobacterium* was previously noted in dogs with multicentric lymphoma,^14^ decreased faecal *C. hiranonis* has not been described in dogs with either multicentric or GI lymphoma. *C. hiranonis* is a key bacterium in bile acid metabolism due to 7 α‐dehydroxylating activity. Decreased faecal *C. hiranonis* has been documented in other chronic enteropathies (CE)^10^ and is associated with decreased faecal secondary bile acids in dogs with inflammatory bowel disease (IBD).^15^ Decreased faecal *C. hiranonis* could suggest bile acid dysregulation in dogs with multicentric lymphoma. However, further exploration through metabolome profiling is needed. Other pre‐treatment microbiome abnormalities that have been described in dogs with multicentric or GI lymphoma, including increased abundances of the Eubacteriaceae family^16^ and *Streptococcus spp* ^14^ and decreased *Faecalibacterium* and *Turicibacter*,^14^ were not observed. Reasons for differences in microbiome taxa shifts are unclear. There could be different mechanisms for dysbiosis in dogs with GI versus non‐GI lymphoma, promoting proliferation or loss of different species. In addition, both the study described here and previous study of dogs with multicentric lymphoma have small populations, so a different dysbiosis pattern could be observed across a larger population. Furthermore, effects of diet and geographic region, which have the highest impact of any variables on the GI microbiome,^17^, ^18^, ^19^, ^20^ cannot be excluded. It is also possible that GI involvement was not completely excluded as a cause of dysbiosis in this population, as only two dogs had abdominal ultrasound performed, and GI biopsies were not performed on any dog. However, no dogs had diarrhoea or vomiting at the time of diagnosis, consistent with previous work.^14^

There were observed effects of treatment group on the GI microbiome in this population. While baseline log DNA/gram faeces were higher in the probiotic group, probiotic administration resulted in higher total bacteria at several other time‐points (days 7 and 24) versus placebo. Decreased faecal total bacteria have been associated with canine CE,^10^ and there are several possible explanations for the differences between groups in this study. The increase in dogs receiving probiotics was likely, in part, due to higher *Streptococcus* and *E. coli*. Increased faecal *Streptococcus* may be secondary to probiotic administration, as *Streptococcus thermophiles* was a supplemented bacteria strain. In contrast*, E. coli* was not directly supplemented. Increased faecal *E. coli* may have been secondary to other bacterial population shifts or metabolome alterations creating a favorable environment for *E. coli* growth. Increased faecal *E. coli* is associated with uncontrolled IBD^10^ and GI lymphoma^16^ in dogs, and increased Proteobacteria, the phylum containing *E. coli*, has been documented in people following chemotherapy.^21^ Chemotherapy alone did not appear to affect faecal *E. coli* in these dogs; however, a combined effect of chemotherapy and probiotics cannot be excluded. It is worth noting that several dogs receiving probiotics, but not the placebo, received amoxicillin clavulanate due to chemotherapy‐induced neutropenia. This antibiotic commonly induces dysbiosis in dogs, including increased abundance and antimicrobial resistance of faecal *E. coli*.^22^, ^23^, ^24^ Therefore, concurrent antibiotics could also account for the increased *E. coli* observed in dogs receiving probiotics. While there were no statistically significant differences in other bacterial taxa, *C. hiranonis* tended to decrease during the study in dogs receiving the placebo and increase in dogs receiving the probiotic. Increasing faecal *C. hiranonis* has been observed following treatment of IBD in dogs and is associated with an improved faecal bile acid profile.^15^ Changes in faecal *C. hiranonis* abundance have also been noted in dogs with acute hemorrhagic diarrhea syndrome following supplementation of the same probiotic.^25^ As metronidazole has been demonstrated to decrease *C. hiranonis* abundance, and only dogs in the placebo group received metronidazole,^26^ this difference cannot be definitively attributed to the probiotic. Several findings associated with probiotic administration, including increased faecal total bacteria, lack of antibiotic‐associated diarrhoea in dogs receiving both probiotics and antibiotics and trends in *C. hiranonis* abundance and DI, suggest positive impact of probiotic administration. While these findings were not all statistically significant, results support expansion of this study to determine whether effects are observed in a larger population. Further faecal analyses, including species‐specific PCR, broad 16S rRNA profiling and metabolome analysis, would be needed to confirm that increased *Streptococcus* abundance was secondary to supplementation and to evaluate probiotic effects on faecal bile acids, respectively.

In addition to treatment group impact on the microbiome, chemotherapy impacted the microbiome at several time‐points. Specifically, faecal *Streptococcus* increased following the second dose of vincristine, regardless of treatment group. While this has not been previously documented in dogs following chemotherapy administration, *Streptococcus* abundance increases in dogs with IBD, suggesting a possible negative impact of chemotherapy.^10^, ^27^ *Streptococcus spp*. are classified within the Firmicutes phylum, which has shown decreased abundance following chemotherapy in people^21^; however, genus and species‐level analyses would be needed to determine whether chemotherapy‐induced microbiome shifts in dogs parallel what is observed in people, as there are multiple species within this phylum. Additional phylum‐level shifts noted in people following chemotherapy include decreased Actinobacteria and increased Proteobacteria^21^; changes in these taxa were not suggested by the targeted analysis in this study.

There are several limitations to this study. First, staging was not complete in all dogs, and different stages and lymphoma classifications (e.g., B versus T cell) were represented in this population. While there could be different dysbiosis manifestations among lymphoma stages or classifications that could impact effects of probiotic therapy, there was no difference in disease severity between treatment groups (i.e., all dogs had high‐grade lymphoma). Differences in dysbiosis patterns among stages and classifications of lymphoma would be a goal for future study. Some dose delays occurred due to neutropenia. While rechecks were performed in all dogs immediately prior to the next chemotherapy dose, not all rechecks were performed on exactly the same day. Ideally, a standard recheck would have been performed 1 week after chemotherapy and then a subsequent recheck prior to the next chemotherapy dose in these dogs. However, this was not feasible with client‐owned dogs, and given the lack of time‐effect on any evaluated parameter, likely had little impact on the analyses. There was variation in faecal collection method between in‐hospital and at‐home samples. However, there is no known impact of short‐term storage at room temperature, refrigeration or overnight shipping of faecal samples.^10^, ^28^, ^29^ Some dogs received ancillary treatments that could impact the GI microbiome, as well as clinical GI signs. These treatments were given for either chemotherapy‐induced neutropenia (amoxicillin clavulanate) or chemotherapy‐related GI effects (metronidazole). While ancillary treatments confound the microbiome changes, it was not deemed ethical to withhold standard‐of‐care treatment from dogs experiencing GI side‐effects or neutropenia. All dogs receiving amoxicillin clavulanate were in the probiotic group. As no dogs in that group experienced adverse GI effects, antibiotic administration likely had little impact on observed clinical signs and if anything, might suggest a protective probiotic effect. This study was not designed to assess probiotic impact on antibiotic‐induced side effects, but a positive effect would be consistent with other studies.^30^, ^31^, ^32^ Lastly, microbiome analysis was not comprehensive but focused on taxa that are altered in dogs with acute and CE, precluding alpha and beta diversity assessment. It is possible that either chemotherapy or probiotics impacted bacterial populations that were not evaluated or overall GI microbiome diversity.

## CONCLUSIONS

Multicentric lymphoma appears associated with GI microbiome dysbiosis in dogs, with alterations in bacteria that could result in bile acid dysregulation. Probiotics were well‐tolerated in this population of dogs, suggesting safety of administration, and tended to decrease episodes of diarrhoea. This study supports further exploration of probiotic benefits on chemotherapy‐induced GI toxicity, as well as the GI metabolome and microbiome diversity.

## FUNDING INFORMATION AND CONFLICT OF INTEREST

This study was supported by the Mark Derrick Canine Research Fund at Kansas State University. The authors declare no conflict of interest.

## AUTHOR CONTRIBUTIONS

Study design, study participant enrollment, data analysis, drafting and revision of the article and final approval of the submitted version: Maria C. Jugan, Raelene M. Wouda and Mary Lynn Higginbotham. Study participant rechecks: Raelene M. Wouda and Mary Lynn Higginbotham. Maria C. Jugan is guarantor.

## Supporting information

Supporting information

TableS1
