# Supplementary material for: Preliminary evaluation of probiotic effects on gastrointestinal signs in dogs with multicentric lymphoma undergoing multi‐agent chemotherapy: A randomised, placebo‐controlled study
Source: Vet Rec Open. 2021 Mar 29;8(1):e2. doi: 10.1002/vro2.2 (PMC8109851; doi:10.1002/vro2.2)
Supplement: Supplementary file 1 — Supporting information [file VRO2-8-e2-s001.pdf]

Recheck Visit Client Questionnaire:

Canine Clinical Signs Score[1]

1. How do you rate your dog's attitude/activity level?
    A. Normal
    B. Slightly decreased but acceptable
    C. Moderately decreased (some activity but to eat, drink, urinate/defecate in acceptable areas)
    D. Severely decreased (reluctant to move but able; does not move to urinate/defecate in acceptable areas; eats if food is offered in place).
    E. Does not move (must be force-fed, needs help to stand/walk)

2. How do you rate your dog's appetite?
    A. Normal
    B. Slightly decreased (3/4 or more of normal; will still eat normal dog food)
    C. Moderately decreased (1/2-3/4 of normal; needs enticement or treats/novel food)
    D. Severely decreased (<1/2 of normal to absent; little to no interest in any food source)

If decreased, how many days? ___________

3. How often does your dog vomit?
    A. Never (no vomiting within past four weeks)
    B. 1 time or less per week
    C. 2-3 times per week
    D. >3 times per week
        If >3 times per week:
        1. <3 times in 24 hours
        2. 3-10 times in 24h/<5 in 48 hours
        3. Multiple episodes lasting >48 hours

4. How would you describe any signs of nausea?
    A. Decreased appetite but no change in eating habits
    B. Salivation or lip-smacking (<3 days)
    C. Salivation or lip-smacking (>3-5 days)
    D. Salivation or lip-smacking (>5 days)
    E. No signs of nausea

5. How would you describe your dog's stool?
    A. Normal (formed stool; able to be picked up)
    B. Slightly soft; presence of mucus or blood
    C. Very soft but still solid; with or without mucus or blood
    D. Liquid diarrhea; with or without mucus or blood

Presence of blood? _______________ Bright red or black? _____________

6. What is the frequency of your dog's defecation?
    A. Normal
    B. 2-3 times per day
    C. 4-5 times per day
    D. >5 times per day

7. How would you describe your dog's weight trends?
    A. No change in weight or weight gain
    B. Mild weight-loss (<5% body weight)
    C. Moderate weight-loss (5-10% body weight)
    D. Severe weight-loss (>10% body weight)

Please estimate the total pounds lost: ________lb__and period of time: __________weeks/months

8. Any trouble swallowing? ______________

9. Any flatulence? _______________

Please answer the following questions:

1. Have you noticed any skin changes (e.g. rash, hair loss, redness, bruising, color change) or itching? Please describe ____

4. What medications is your dog taking? ____

5. What food does your dog eat? ____

6. What treats does your dog receive? ____

7. What supplements does your dog receive (e.g. glucosamine, probiotics)? ____________________________

8. Any weakness/collapse since last visit? ____________________

1- Veterinary cooperative oncology group. Common terminology for adverse events (VCOG-CTCAE) following chemotherapy or biological antineoplastic therapy in dogs and cats v1.1. *Vet Comp Oncol*. 2016; 14: 417-446.
