# Supplementary material for: Preliminary evaluation of probiotic effects on gastrointestinal signs in dogs with multicentric lymphoma undergoing multi‐agent chemotherapy: A randomised, placebo‐controlled study
Source: Vet Rec Open. 2021 Mar 29;8(1):e2. doi: 10.1002/vro2.2 (PMC8109851; doi:10.1002/vro2.2)
Supplement: Supplementary file 2 — TableS1 [file VRO2-8-e2-s002.pdf]

Supplement 2. Individual dog findings and lymphoma characteristics of 10 dogs undergoing CHOP-based chemotherapy for multi-centric lymphoma and receiving either probiotic or placebo. Gastrointestinal severity scores are expressed as the average score per time period. Anatomic area in bold under disease localization denotes sites of cytologic or histopathologic diagnosis of lymphoma. CD, cluster of differentiation; FI, female intact; FS, female spayed; Ig, immunoglobulin; MC, male castrated; MHC, major histocompatibility complex; MI male intact

Dog	Treatment	Age (years)	Breed	Sex	Disease Localization - Site of Cytologic or Histopathologic Diagnosis & Physical Examination Abnormalities	Thoracic Radiographs	Abdominal Ultrasound	Flow	B vs T Cell	Cell Size
**1**	Probiotic	13	Jack Russell Terrier	FS	**Generalized peripheral lymphadenopathy**; Mild hepato-/splenomegaly	No abnormalities	Not assessed	CD45+: 99%<br>CD21+: 95%<br>Ig+: 91%<br>MHCII: 52%	B cell	Intermediate
**2**	Placebo	9	Labrador Retriever	FS	**Generalized peripheral lymphadenopathy**; Mild hepatomegaly	Not assessed	Not assessed	CD45+: 100%<br>CD21+: 93%<br>Ig: 98%<br>MHCII: 98%	B cell	Large
**3**	Placebo	9	Heeler	MC	**Hepatic**; **Abdominal effusion**; Mild prescapular lymph node enlargement	Not assessed	Diffuse hepatomegaly with hypoechogenicity and mottled appearance to parenchyma; Diffusely hypoechoic intra-abdominal lymph nodes (0.8-3.6 cm); Moderate	CD45+: 99%<br>CD5+: 60%<br>CD4+: 32%<br>CD4+CD8+: 55%	T cell	Intermediate

							volume anechoic peritoneal effusion			
**4**	Probiotic	8	Scottish Terrier	FS	**Generalized peripheral lymphadenopathy**	Not assessed	Not assessed	Not assessed	Not assessed	Large
**5**	Placebo	6	Labrador Retriever	MC	**Generalized peripheral lymphadenopathy**	No abnormalities	Hepatic hypoechogenicity; Mottled spleen; Intra-abdominal lymphadenopathy (2.4-3.2 cm)	Not assessed	Not assessed	Large
**6**	Placebo	12	Miniature Poodle	MC	**Generalized peripheral lymphadenopathy**	Not assessed	Not assessed	Not assessed	B cell	Intermediate
**7**	Probiotic	7	Golden Retriever	FI	**Generalized peripheral lymphadenopathy**	Not assessed	Not assessed	Not assessed	T cell	Intermediate
**8**	Probiotic	4	Golden Retriever	FI	**Generalized peripheral lymphadenopathy**	Not assessed	Not assessed	Not assessed	T cell	Intermediate
**9**	Placebo	14	Jack Russell Terrier	FS	**Generalized peripheral lymphadenopathy**	Not assessed	Not assessed	Not assessed	B cell	Large
**10**	Probiotic	4	Australian Shepherd	FS	**Generalized peripheral lymphadenopathy**; Mild hepatomegaly	Not assessed	Not assessed	CD45+: 99%<br>CD21+: 96%<br>Ig: 88%<br>MHCII: 99%	B cell	Large
